# Heme Oxygenase-1 Expression in Dendritic Cells Contributes to Protective Immunity against Herpes Simplex Virus Skin Infection

**DOI:** 10.3390/antiox12061170

**Published:** 2023-05-29

**Authors:** Eduardo I. Tognarelli, Luisa F. Duarte, Mónica A. Farías, Felipe A. Cancino, Nicolás Corrales, Francisco J. Ibáñez, Claudia A. Riedel, Susan M. Bueno, Alexis M. Kalergis, Pablo A. González

**Affiliations:** 1 Millennium Institute on Immunology and Immunotherapy, Departamento de Genética Molecular y Microbiología, Facultad de Ciencias Biológicas, Pontificia Universidad Católica de Chile, Santiago 8331150, Chilefacancino@uc.cl (F.A.C.); claudia.riedel@unab.cl (C.A.R.);; 2 Millennium Institute on Immunology and Immunotherapy, Departamento de Ciencias Biológicas, Facultad de Ciencias de la Vida, Universidad Andrés Bello, Santiago 8370133, Chile; 3 Departamento de Endocrinología, Facultad de Medicina, Escuela de Medicina, Pontificia Universidad Católica de Chile, Santiago 8320000, Chile

**Keywords:** heme oxygenase-1, dendritic cells, adaptive response, skin infection, antiviral drug, herpes simplex virus, Th17, Treg, Treg/Th17

## Abstract

Herpes simplex virus type 1 (HSV-1) and type 2 (HSV-2) infections are highly prevalent in the human population and produce mild to life-threatening diseases. These viruses interfere with the function and viability of dendritic cells (DCs), which are professional antigen-presenting cells that initiate and regulate the host’s antiviral immune responses. Heme oxygenase-1 (HO-1) is an inducible host enzyme with reported antiviral activity against HSVs in epithelial cells and neurons. Here, we sought to assess whether HO-1 modulates the function and viability of DCs upon infection with HSV-1 or HSV-2. We found that the stimulation of HO-1 expression in HSV-inoculated DCs significantly recovered the viability of these cells and hampered viral egress. Furthermore, HSV-infected DCs stimulated to express HO-1 promoted the expression of anti-inflammatory molecules, such as PDL-1 and IL-10, and the activation of virus-specific CD4^+^ T cells with regulatory (Treg), Th17 and Treg/Th17 phenotypes. Moreover, HSV-infected DCs stimulated to express HO-1 and then transferred into mice, promoted the activation of virus-specific T cells and improved the outcome of HSV-1 skin infection. These findings suggest that stimulation of HO-1 expression in DCs limits the deleterious effects of HSVs over these cells and induces a favorable virus-specific immune response in the skin against HSV-1.

## 1. Introduction

Herpes simplex virus type 1 (HSV-1) and type 2 (HSV-2) infections are highly prevalent worldwide [1], with both viruses establishing lifelong persistent infections in humans and sporadically reactivating, which can translate into virus shedding and recurrent disease, mainly in the skin [2]. Due to their impact over human health, considerable efforts have been invested in improving the available treatments against these viruses, as well as preventing infection [3,4,5]. Nevertheless, at present, there are no vaccines available against HSV-1 or HSV-2.

HSVs can effectively infect a wide array of cell types and cause productive infection in multiple tissues [2,6]. Importantly, these viruses can infect immune cells and modulate their functions to interfere with the host antiviral responses [7]. Indeed, HSVs hamper innate immune antiviral responses, such as type-I interferon production, and can hinder the activation of an effective adaptive immune response [8,9,10].

Dendritic cells (DCs) are immune cells that play important roles in initiating and regulating antiviral responses in the host by sensing, processing, and presenting virus-derived antigens to T cells [11], which in turn can promote B cell activation and the production of effector antibodies [11,12]. HSVs have been extensively reported to hinder DC function in multiple ways [7,13], such as reducing antigen presentation on MHC-I molecules [14,15], decreasing the expression of co-stimulatory molecules that support T cell activation [9,16], interfering with autophagosome function and limiting their migration to lymph nodes where they activate T cells [17,18]. Furthermore, upon replication in DCs, HSVs induce apoptosis in these cells [8,10,13,19,20,21].

Previously, we reported that the pharmacological induction of the host enzyme heme oxygenase-1 (HO-1) in epithelial cells and neurons blocks the replication of HSV-2 [22]. HO-1 is induced in mammal cells in response to stress, such as hypoxia, or pathological conditions that release porphyrins (cofactors) that, if not correctly processed by the cell, leads to oxidative stress [23,24]. HO-1 catalyzes the conversion of iron-containing porphyrin groups, such as heme, into three products: ferrous iron (Fe^2+^), carbon monoxide (CO), and biliverdin [25], the latter being rapidly converted into bilirubin by biliverdin reductase [26]. Importantly, all these products have cytoprotective effects for the cell, such as reducing apoptosis [25,27].

Because the impact of HO-1 expression in DCs has not been assessed in the context of HSV infection and the fact that this enzyme exerts significant immunomodulatory effects over these cells, we sought to evaluate whether the induction of HO-1 expression in DCs has an antiviral effect against HSVs in these cells. Furthermore, we sought to assess the contribution of HO-1 expression over the activation of virus-specific T cells in vitro and in vivo by HSV-infected DCs, and its impact on HSV-1 infection in an HSV-1 skin infection model. For this, we used protoporphyrin analogs, namely cobalt protoporphyrin-IX (CoPP), which promotes HO-1 expression [28], and tin protoporphyrin-IX (SnPP), which inhibits the activity of this enzyme [29]. Additionally, we induced HO-1 expression in DCs differentiated from the bone marrow of pli-rtTA-tetO-HO-1 (herein tTA-HO-1) transgenic mice, which express HO-1 in MHC-II-expressing cells in response to doxycycline (Dox) added to the cell cultures or to the drinking water of these animals [27].

## 2. Materials and Methods

### 2.1. Mice

8-week-old female C57BL/6J mice were obtained from Jackson Laboratory (Bar Harbor). pli-rtTA-tetO-HO-1 mice (tTA-HO-1), which express HO-1 in MHC-II^+^ cells in response to doxycycline (Dox) added to the culture media or to drinking water of the corresponding animals, were kindly shared by Dr. George Kollias from the Biomedical Sciences Research Centre “Alexander Fleming” in Greece. All mice were maintained at the animal facility at the Pontificia Universidad Católica de Chile and handled according to the guidelines of the Institutional Ethics Committee at the same university (approved protocols CBB-201/2013 and CEC 180821026). The gBT-I transgenic mouse strain, which encodes an HSV-specific CD8^+^ T cell receptor (TCR) that recognizes MHC-I/viral peptide gB_498–505_, was shared by Dr. Francis Carbone [30] and provided by Dr. Akiko Iwasaki from Yale University, USA. The gDT-II transgenic mouse strain encoding an HSV-specific CD4^+^ TCR that recognizes MHC-II/viral peptide gD_290–302_ was also shared by Dr. Francis Carbone [31] and provided by Dr. David Taylor from the University of Melbourne, Australia.

### 2.2. Virus Propagation

Vero cells (ATCC CCL-81) were used to propagate and titer HSV-1 KOS (ATCC VR-733), HSV-1 K26GFP [32], HSV-2 (G) [13], and HSV-2 (333) ZAG [33], as previously described [21]. (The viruses were kindly provided by Drs. Betsy Herold and Natalia Cheshenko from the Albert Einstein College of Medicine, USA.)

### 2.3. Viability and Maturation of HSV-Infected DCs

Bone-marrow-derived DCs (BMDCs) were obtained from the bone marrow of C57BL/6J mice as previously described (WT DCs) [34]. 6 days after the extraction of the bone marrow and culturing with GM-CSF, the DCs were treated with 50 µM cobalt protoporphyrin-IX (CoPP) dissolved in sodium hydroxide (NaOH 0.1 M); 50 µM tin protoporphyrin-IX (SnPP) dissolved in NaOH; 60 µM carbon monoxide-releasing molecule-2 (CORM-2; Tricarbonyldichlororuthenium(II)) in DMSO; or 60 µM inactivated CORM-2 (iCORM-2) with 0.1 M NaCl for 2 h; then neutralized with 0.1 M NaOH [22], or an equivalent volume of vehicle NaOH 0.1 M for 6 h. In parallel, DC cultures derived from tTA-HO-1 mice, which encode an inducible HO-1 gene under the control of a tetracycline promoter, were treated in vitro with doxycycline (Dox, 1.5 µg/mL) or PBS vehicle for 16 h. Afterward, DCs were infected with HSV-1 or HSV-2 at a MOI of 3 for 1 h at 37 °C. Supernatants were then removed, and the cells were washed with culture media. Then, the cells were cultured with the corresponding treatments in culture media (CoPP, SnPP, CORM-2, iCORM-2, or vehicle for WT DCs; Dox or PBS for transgenic DCs). For assessing cell viability, the Zombie NIR dye (Biolegend, San Diego, CA, USA) was used. Cells were also stained with antibodies against CD11c and MHC-II (Biolegend, San Diego, CA, USA). The GFP reporter gene was used as a readout of infection in experiments using viruses HSV-1 K26GFP or HSV-2 (333) ZAG. Cells were fixed after the corresponding time-points indicated using 2% paraformaldehyde (PFA) and analyzed in a FACSCANTO II flow cytometer (BD Biosciences, San Jose, CA, USA). For assessing the maturation of BMDCs, cells were stained with antibodies against MHC-I, MHC-II, CD80, CD86, PDL-1, PDL-2, and/or OX40L (Biolegend, San Diego, CA, USA) and analyzed using a FACSVia flow cytometer (BD Biosciences, San Jose, CA, USA). Representative dot plots were included in Figure S4. For assessing cytokines secreted by BMDCs after the different treatments, ELISA was performed using antibodies against IL-6, IL-10, IL-12, IL-23, IL-1β, and TGF-β (Biolegend, San Diego, CA, USA), and cell supernatants were recovered 24 h after treatment, as previously reported [13]. Recombinant cytokines (PeproTech, Cranbury, NJ, USA) were used as standards.

### 2.4. Caspase-3 Activity Assay and HSV Replication in Infected DCs

We measured caspase-3 activity in DCs to complement the analysis of cell viability performed by flow cytometry using Zombie NIR dye (Biolegend, San Diego, CA, USA). Caspase-3 has been previously reported to be activated in HSV-infected DCs [13,35]. Briefly, 3 × 10^5^ DCs were seeded and infected with HSV-1 KOS or HSV-2 G for 1 h at MOI 3. The culture media was then replaced with fresh media. 24 hpi, DCs were collected and centrifuged at 400× *g* for 5 min at 4 °C. The pellet was resuspended in 100 µL of lysis buffer, incubated at 4 °C for 15 min and dispensed in a 96-well black plate containing 100 µL of the Ac-DEVD-AFC (Cayman, Ann Arbor, MI, USA) caspase-3 substrate. Then, the mixture was incubated for 1 h and analyzed at 400/505 nm emission using a Cytation 5 Multi-Mode Reader (BioTek, Winooski, VT, USA). DC infection with HSV-1 KOS and HSV-2 G was assessed by PFU assays in triplicates, adding supernatants from infected-DCs over Vero cell monolayers seeded 24 h prior in flat-bottom 96-well plates. Extraction of total DNA from infected cells was carried out as previously described [4] and qPCR was performed using 500 ng of DNA per reaction, as previously described [13], using an Applied Biosystems StepOnePlus thermocycler (Thermofisher, Waltham, MA, USA) [36]. Total protein extraction was performed using RIPA buffer (Cell Signaling, Danvers, MA, USA). 30 µg of protein extract was loaded in a 10% polyacrylamide gel (BioRad, Hercules, CA, USA) and transferred onto a 0.45 µm nitrocellulose membrane. The membrane was then blocked with BSA 5% and then incubated with anti-gB (1:1000; Virusys, Taneytown, MD, USA), anti-HO-1 (1:1000, clone HO-1-1, Thermofisher, Waltham, MA, USA), and HRP-anti-β-actin (1:5000; clone F21-1, GenScript, Piscataway, NJ, USA) to determine the viral protein expression at 24 hpi. The Western blot bands were analyzed using ImageJ software ver.2.1.0 [37].

### 2.5. DC-T Cell Antigen Presentation Assays

WT DCs were treated with 50 µM of CoPP, SnPP; 60 µM of CORM-2, iCORM-2, or an equivalent volume of vehicle (NaOH) for 6 h; or transgenic DCs were treated with 1.5 µg/mL of Dox or PBS for 16 h, then infected at an MOI of 3 with HSV-1 KOS or HSV-2 G for 1 h at 37 °C. Then, the supernatants were removed, washed with culture media, and incubated with the corresponding treatments. DCs were collected 6 h later and co-cultured with 1 × 10^5^ HSV-specific T cells/well. T cells, either CD8^+^ gBT-I or CD4^+^ gDT-II, were purified from the spleens of transgenic mice using negative-selection kits for T cells (MiltenyiBiotec, Bergisch Gladbach, Germany). Uninfected DCs and DCs pulsed with gD_290–302_ or gB_498–505_ peptides were used as controls, as previously described [13]. T cell activation and differentiation were determined by measuring IL-2 and IFN-γ using ELISA. Additionally, for CD4^+^ T cells, IL-4 and IL-17 were measured in the supernatants [34]. Cell viability, CD4, CD8, CD25, and CD71 surface markers were assessed by flow cytometry (BioLegend, San Diego, CA, USA). To further determine CD4^+^ T cell differentiation, antibodies against the surface expression markers OX40 and PD-1 were used, while antibodies against FoxP3 and RORγt were used for intracellular staining (Biolegend, San Diego, CA, USA), followed by FACS analysis.

### 2.6. HSV Skin Infection and T Cell Activation In Vivo

For the in vivo HSV skin infection assays, DCs were first treated in vitro with 50 µM of CoPP or SnPP, or an equivalent volume of vehicle (NaOH) for 6 h, and then infected at an MOI 3 with HSV-1 KOS for 1 h at 37 °C. After 6 h of treatment, 5 × 10^5^ treated DCs were inoculated intraperitoneally in 50 μL volume into 6 weeks old C57BL/6J mice. These mice were then anesthetized with an intraperitoneal injection of a ketamine/xylazine solution, and skin infection was performed as previous described [38]. Briefly, the right flank of each mouse was depilated and shaved using VEET^®^ depilating agent. The following day, the epidermal layer of the skin was exposed by abrasion with a nail file. 24 h later, mice were infected with 10^6^ PFU of HSV-1 (KOS), or with mock (Vero cell preparations equivalent to those for obtaining Vero cell-derived infectious HSV) in 10 μL volume. Vehicle treatments were used as controls. HSV-1 disease progression was followed for 13 days by evaluating the clinical score of zosteriform lesions as follows: (1) erythema or primary lesion, (2) slight erythema/oedema and distant zosteriform lesions, (3) ulceration and oedema, increased epidermal spread, and (4) hind limb paralysis [39]. At days 4 and 10 post infection, the animals were euthanized and skin sections of a 1 cm^2^ area were recovered and treated with collagenase-D for 30 min at 37 °C, followed by physical disaggregation using a mortar, from which viral titers were determined over Vero cells and viral genome loads by qPCR, as described above. To assess the activated T cell populations in vivo in HSV-infected mice, virus-specific gBT-I CD8^+^ and gDT-II CD4^+^ naïve T cells were stained with Vybrant™ Dil cell-labeling dye (LifeTechnologies, Waltham, MA, USA) and transferred intravenously into mice 24 h prior to receiving CoPP-, SnPP-, or vehicle-treated and HSV-infected DCs, i.e., 48 h prior to skin infection. 4 and 10 days after skin infection with HSV-1 KOS, T cell analyses were performed using the following surface markers: CD4, CD8, CD25, CD71, FoxP3, GATA-3, and RORγt (BioLegend, San Diego, CA, USA) by FACS in a BD LSRFortessa X-20 flow cytometer (BD Biosciences, San Jose, CA, USA). Representative dot plots were included in Figure S5.

### 2.7. Statistical Analyses

Statistical analyses between experimental groups were assessed either by unpaired Student’s *t*-test (bar graphs, two groups), one-way analysis of variance (ANOVA) with Tukey’s multiple comparison test (three or more groups), or Kruskal–Wallis with Dunnett’s multiple comparison test (two independent variables), with a confidence interval of 95%, as indicated in each figure, using GraphPad Prism (GraphPad Prism 6 Software ver.6.01).

## 3. Results

### 3.1. HO-1 Expression in HSV-Infected DCs Enhances Cell Viability and Hampers Viral Particle Egress without Affecting Viral Genome Replication or Transcription

**Figure 1:**
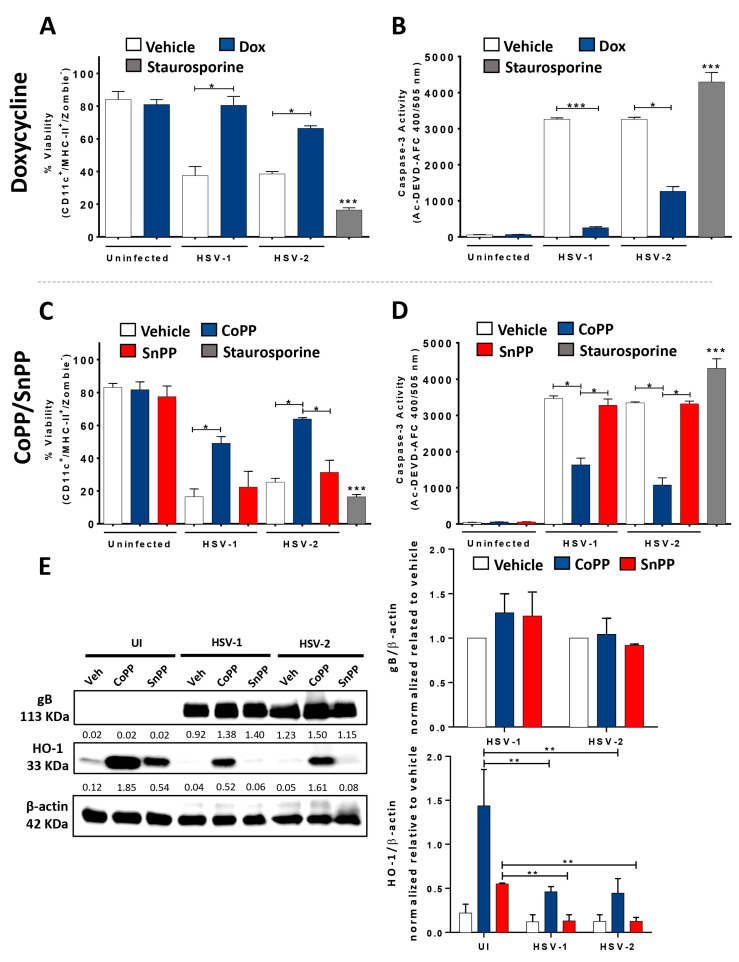

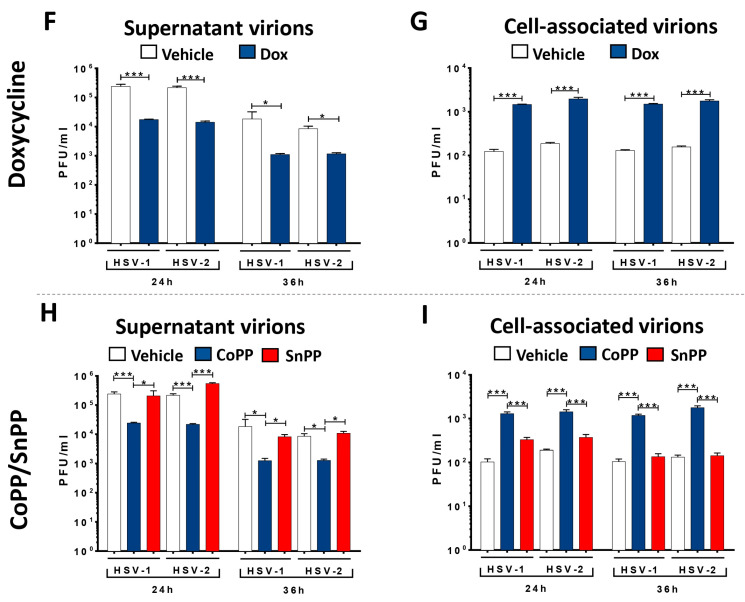
HO-1 expression in DCs enhances the viability of HSV-infected DCs and hampers viral particle release from infected cells. (**A**) DC viability measured by FACS (in CD11c^+^, MHC-II^+^ (I-A^b^), Zombie^-^ cells) at 24 h post-infection (hpi) with HSV-1 KOS or HSV-2 (333) ZAG in DCs previously treated with doxycycline (Dox, 1.5 μg/mL) for 16 h. (**B**) Caspase-3 activation in Dox-treated HSV-1 or HSV-2-infected DCs at 24 hpi was measured through fluorescence derived from substrate Ac-DEVD-AFC. (**C**) Viability measured at 24 hpi by FACS in HSV-1- or HSV-2-infected DCs previously treated with CoPP or SnPP (50 µM) for 6 h. (**D**) Caspase-3 activity in CoPP-treated DC cultures 24 hpi. (**E**) Left: Western blot analyses for gB (113 KDa) and HO-1 (33 KDa) from total protein samples obtained from Dox-, CoPP- or SnPP-treated HSV-infected DCs, cultured in vitro, 18 h after infection, with their respective vehicle-treated and uninfected controls. Right: densitometry analyses for gB (upper panel) and HO-1 (lower panel) expression relative to β-actin. (**F**) Quantification of infectious viral particles released into the supernatants of DC cultures (PFU/mL) at 24 and 36 hpi with HSV-1 or HSV-2, previously treated for 16 h with doxycycline (Dox, 1.5 µg/mL). (**G**) Cell-associated infectious virus particles in HSV-infected and Dox-treated DCs obtained from cell lysates at 24 and 36 hpi. (**H**) Quantification of infectious viral particles released into the supernatants of DC cultures (PFU/mL) at 24 and 36 hpi with HSV-1 or HSV-2, previously treated for 6 h with CoPP or SnPP (50 µM) at 24 and 36 hpi. (**I**) Cell-associated infectious virus particles in HSV-infected and CoPP- or SnPP-treated DCs. Veh: vehicle. Staurosporine was used as a cell death positive control. Statistical analysis: Kruskal–Wallis and Dunnett’s multiple comparison (*** *p* < 0.001, ** *p* < 0.01, * *p* < 0.05).

To assess the contribution of HO-1 expression over the integrity and phenotype of HSV-infected DCs, first, we evaluated the viability of bone-marrow-derived DCs (BMDCs) treated to express HO-1 and infected with either, HSV-1 or HSV-2 at 24 h post-infection (hpi). We observed by flow cytometry that pli-rtTA-tetO-HO-1-transgenic-mice-derived DCs stimulated with doxycycline (Dox), which promotes HO-1 expression, significantly reduced both HSV-1- and HSV-2-mediated cell death. This resulted in approximately 80–60% of viable cells, whereas vehicle-treated and HSV-infected DCs displayed significantly lesser viability (<40%) (Figure 1A). These results were consistent with reduced activated caspase-3 in Dox-treated HSV-infected DCs compared to HSV-infected DCs that were not treated with Dox, which also suggests enhanced cell viability in the DCs treated to express HO-1, as caspase-3 activity has been related to HSV-induced apoptosis in these cells (Figure 1B) [13,35].

Similarly, inducing HO-1 expression with CoPP in HSV-infected DC cultures elicited 50–65% viable cells, compared to the less than 30% viable DCs when treated with the HO-1 antagonist SnPP and infected with HSV (Figure 1C). Activated caspase-3 levels were also significantly reduced in CoPP-treated and HSV-infected DCs as compared to SnPP- or vehicle-treated and HSV-infected DCs (Figure 1D). These results suggest that the stimulation of HO-1 expression in DCs positively modulates the viability of HSV-infected DCs, supporting a cytoprotective role for this host factor upon HSV infection.

Next, the expression of the late viral glycoprotein B (gB) and HO-1 in CoPP-treated DCs was assessed by Western blot, wherein we did not observe any notable differences for gB expression between treatments, but we did see increased HO-1 levels in DCs stimulated to express this protein compared to the vehicle-treated or SnPP-treated controls (Figure 1E).

Next, we sought to assess whether HO-1 expression in HSV-infected DCs interferes with the replication cycle of these viruses. Here, we found that the stimulation of HO-1 expression in DCs infected with HSV did not produce any statistically significant differences in viral genome replication between groups, as determined by qPCR (Supplementary Figure S1A,E). Likewise, we found that Dox or CoPP treatments did not alter the expression levels of the early viral mRNA transcript of the *ICP0* gene (RT-qPCR) at 6 or 9 hpi (Supplementary Figure S1B,F).

However, when evaluating the virus yield from HSV-infected DCs stimulated to express HO-1, we found that either Dox or CoPP treatment significantly reduced HSV PFU outputs in the cell supernatants compared to vehicle-treated and HSV-infected DCs (Figure 1F,H). When evaluating the amount of intracellular and cell-associated virions in these cells, we found that HO-1 induction in DCs significantly increased the amount of cell-associated infectious viral particles in these cells (Figure 1G,I). Overall, these results indicate that HO-1 expression in DCs hampers infectious viral particle egress from these cells without affecting viral genome replication, transcription, or viral protein synthesis. Nevertheless, the relative accumulation of cell-associated virions in HO-1-expressing DCs could be due to a delay in the virus replication cycle in these cells, as compared to control cells.

### 3.2. HO-1 Expression in HSV-Infected DCs Promotes an Anti-Inflammatory Phenotype

**Figure 2:**
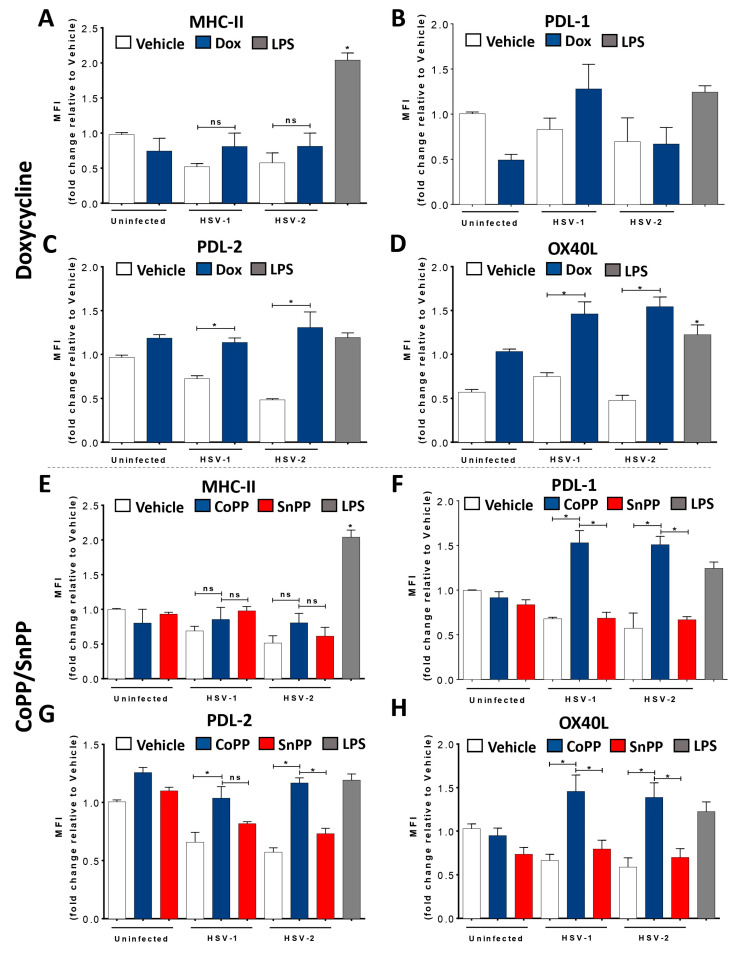

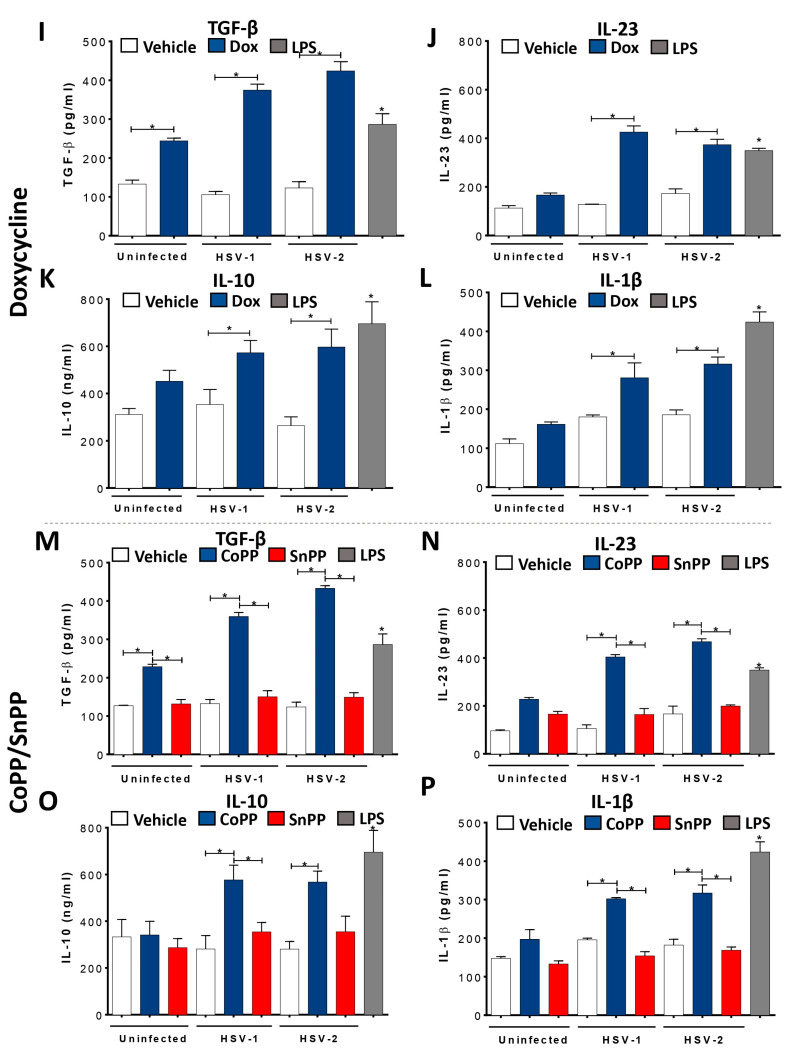
HSV-infected DCs induced to express HO-1 upregulate PDL-1, PDL-2 expression, and IL-10 secretion. (**A**) Surface expression of MHC-II (I-Ab) in DCs measured by FACS (in CD11c^+^, MHC-II^+^ (I-A^b^), Zombie^-^) at 24 hpi with HSV-1 or HSV-2, previously treated for 16 h with doxycycline (Dox, 1.5 μg/mL). Surface expression of (**B**) PDL-1, (**C**) PDL-2 and (**D**) OX40L in HSV-1- or HSV-2-infected and Dox-treated DCs. (**E**) Surface expression of MHC-II in DCs measured by FACS at 24 hpi in HSV-1- or HSV-2-infected DCs previously treated with CoPP or SnPP (50 µM) for 6 h. Surface expression of (**F**) PDL-1, (**G**) PDL-2 and (**H**) OX40L in HSV-1- or HSV-2-infected and CoPP-treated DCs. Detection of (**I**) TGF-β, (**J**) IL-23, (**K**) IL-10 and (**L**) IL-1β secretion in the supernatants of HSV-1- and HSV-2-infected and Dox-treated DCs. Detection of (**M**) TGF-β, (**N**) IL-23, (**O**) IL-10 and (**P**) IL-1β secretion in the supernatants of HSV-1- and HSV-2-infected and Dox-treated DCs. LPS: lipopolysaccharide. Statistical analysis: Kruskal–Wallis and Dunnett’s multiple comparison (* *p* < 0.05).

Next, we evaluated the phenotype of DCs stimulated to express HO-1 and then infected with HSV-1 or HSV-2. Importantly, neither Dox nor CoPP treatments significantly increased or decreased the surface expression of DC maturation markers, such as CD80, CD86, or MHC-I, compared to SnPP- or vehicle-treated HSV-infected DCs (Supplementary Figure S2). Similarly, no significant differences were observed for MHC-II surface expression in Dox-treated or CoPP-treated HSV-infected DCs compared to non-treated HSV-infected transgenic DCs (Figure 2A) or non-treated HSV-infected WT DCs (Figure 2E).

However, the expression of the co-inhibitory molecules PDL-1 and PDL-2 significantly increased on the surface of HSV-infected DCs when treated with the HO-1 inducer Dox (Figure 2B for HSV-1 but not HSV-2, and Figure 2C) or CoPP (Figure 2F,G), although this was somewhat independent of HSV infection for PDL-2 (Figure 2C,G). Importantly, we found different results regarding PDL-1 expression, with significantly increased PDL-1 expression for the CoPP- but not for Dox-treatment (Figure 2B,F). While PDL-1 and PDL-2 are ligands for PD1 in T cells and promote CD4^+^ T cell differentiation into regulatory T cells (Treg) [40], we also found an increase in OX40L expression in DCs induced to express HO-1 (Figure 2D,H). OX40L binds to OX40 on the surface of T cells and is reported to promote their expansion, altogether promoting a Th2 phenotype and inhibiting an increase in Tregs [41,42], although opposing findings are reported in the literature regarding this observation [43,44].

Additionally, we assessed the secretion of IL-6, IL-12 (Supplementary Figure S1), TGF-β, IL-23, IL-10, and IL-1β (Figure 2I–P) in the supernatants of HSV-infected DCs previously treated or untreated with the inducers of HO-1 expression. Overall, TGF-β, IL-23, IL-10, and IL-1β expression were significantly increased in HSV-infected DCs previously treated with Dox or CoPP compared to SnPP- or vehicle-treated HSV-infected DCs (Figure 2I–P).

Importantly, these results suggest that HO-1 induction modulates the outcome of the DC phenotype after HSV infection, with HO-1 expression positively modulating DC viability and restricting virus-mediated cell death. Although no observable canonical maturation profiles were observed, the surface markers detected in these cells and the cytokines secreted into the extracellular media suggest a trend towards the stimulation of CD4 T cells into T helper 17 (Th17) or T reg profiles.

### 3.3. HO-1 Expression in HSV-Infected DCs Promotes CD4^+^ T Cell Activation In Vitro

Based on the findings described above, we sought to assess the effect of the stimulation of HO-1 expression in HSV-infected DCs over the capacity of these cells to activate gD viral antigen-specific transgenic CD4^+^ T cells (gDT-II) and gB viral antigen-specific transgenic CD8^+^ T cells (gBT-I) in co-cultures.

**Figure 3:**
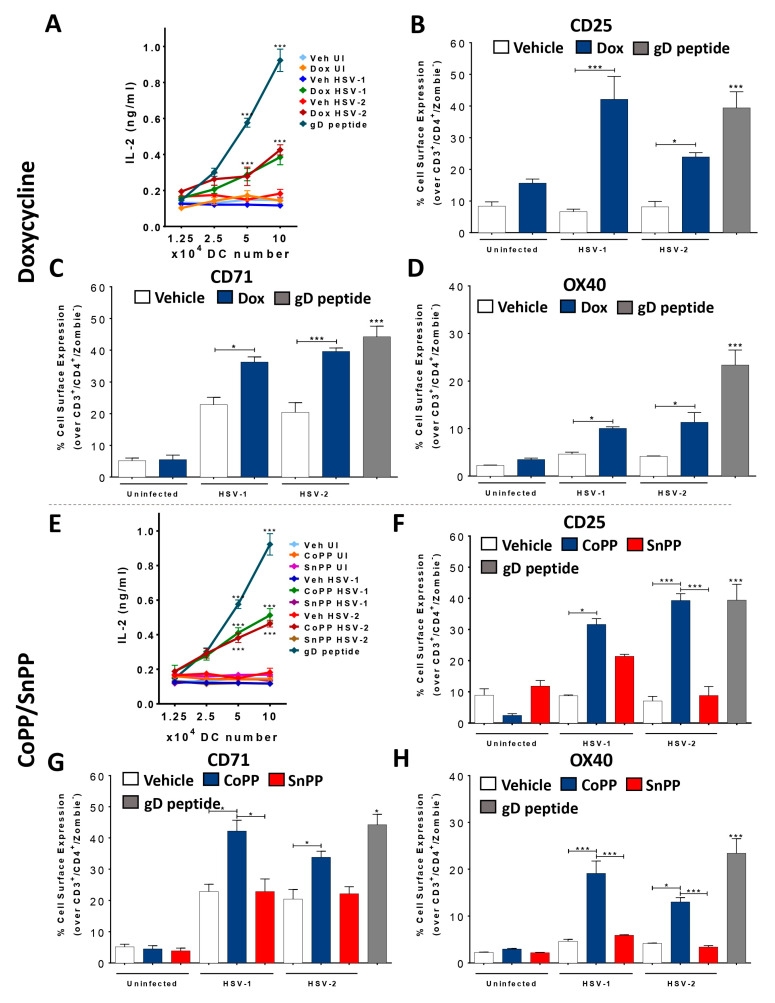

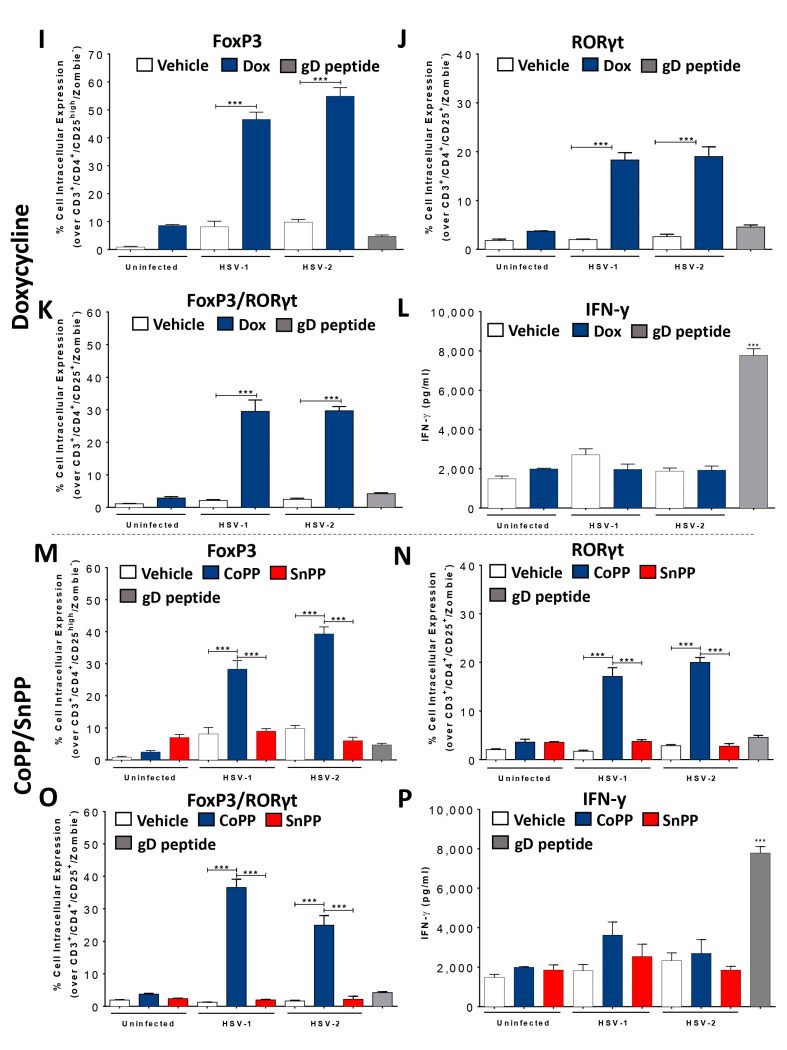

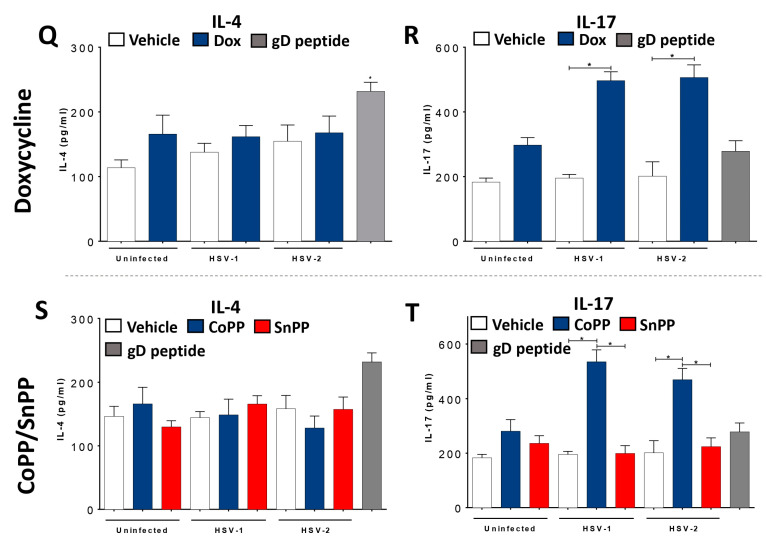
HSV-infected DCs induced to express HO-1 promote the polarization of virus-specific CD4^+^ T cells toward Treg, Th17 and mixed Treg/Th17 phenotypes in vitro. (**A**) IL-2 secretion in supernatants of co-cultures with transgenic virus-specific gDT-II CD4^+^ T cells and HSV-1- or HSV-2-infected and treated DCs, measured by ELISA at 48 hpi. DCs were previously treated for 16 h with doxycycline (Dox, 1.5 μg/mL) or vehicle. (**B**) Surface expression of CD25 in CD4^+^ T cells co-cultured with HSV-infected Dox-treated DCs, measured by FACS (in CD3^+^, CD4^+^, Zombie^-^ cells) 48 hpi. (**C**) Surface expression of CD71 in CD4^+^ T cells. (**D**) Surface expression of OX40 in CD4^+^ T cells. (**E**) IL-2 secretion in the supernatants of co-cultures with CD4^+^ T cells and HSV-infected DCs previously treated for 6 h with CoPP or SnPP (50 µM). (**F**) Surface expression of CD25 in CD4^+^ T cells (CoPP/SnPP). (**G**) Surface expression of CD71 in CD4^+^ T cells (CoPP/SnPP). (**H**) Surface expression of OX40 in CD4^+^ T cells (CoPP/SnPP). (**I**) Intracellular expression (CD3^+^, CD4^+^, CD25^high^, Zombie^-^ cells) of the transcription factor FoxP3 in CD4^+^ T cells co-cultured with Dox-treated DCs. (**J**) Intracellular expression of RORγt in CD4^+^ T cells co-cultured with Dox-treated DCs. (**K**) Intracellular expression of transcription factor FoxP3 and RORγt in CD4^+^ T cells co-cultured with Dox-treated DCs. (**L**) IFN-γ secretion in supernatants of co-cultures with CD4^+^ T cells and Dox-treated DCs. (**M**) Intracellular expression of transcription factor FoxP3 in CD4^+^ T cells (CD3^+^, CD4^+^, CD25^high^, Zombie^-^ cells) co-cultured with CoPP- or SnPP-treated DCs. (**N**) Intracellular expression of RORγt in CD4^+^ T cells co-cultured with CoPP- or SnPP-treated DCs. (**O**) Intracellular expression of transcription factor FoxP3 and RORγt in CD4^+^ T cells co-cultured with CoPP- or SnPP-treated DCs. (**P**) IFN-γ secretion in supernatants of co-cultures with CD4^+^ T cells and CoPP- or SnPP-treated DCs. (**Q**) IL-4 secretion in supernatants of co-cultures with CD4^+^ T cells and Dox-treated DCs. (**R**) IL-17 secretion in supernatants of co-cultures with CD4^+^ T cells and Dox-treated DCs. (**S**) IL-4 secretion in supernatants of co-cultures with CD4^+^ T cells and CoPP- or SnPP-treated DCs. (**T**) IL-17 secretion in supernatants of co-cultures with CoPP- or SnPP-treated DCs. CD4^+^ T were stimulated with gD peptide as a positive control. Statistical analysis: Kruskal–Wallis and Dunnett’s multiple comparison (* *p* < 0.05, *** *p* < 0.001).

Dox treatment in HSV-infected DCs elicited significant IL-2 secretion by virus-specific CD4^+^ T cells in the supernatants, an indication of T cell activation (Figure 3A). Additionally, we observed a significant increase in CD25 expression (the light chain of the IL-2 receptor) on the surface of these virus-specific CD4^+^ T cells by flow cytometry, which also accounts for T cell activation (Figure 3B). Furthermore, HO-1 induction in HSV-infected DCs led to a significant increase in the surface expression of CD71 in CD4^+^ T cells, an early activation marker in these cells that was not observed in co-cultures with SnPP- or vehicle-treated HSV-infected DCs (Figure 3C). DCs treated with CoPP and infected with HSV also elicited a significant increase in IL-2 secretion by T cells (Figure 3E), as well as CD25 (Figure 3F) and CD71 (Figure 3G) surface expression in these cells compared to the control conditions.

Next, we sought to assess the polarization of virus-specific CD4^+^ T cells co-cultured with HO-1-expressing and HSV-infected DCs to better characterize their phenotypes. Because HO-1 expression in DCs mostly relates to Tregs [45,46,47], we assessed Forkhead box protein P3 (FoxP3) in CD4^+^ T cells in the co-cultures (Figure 3). Interestingly, we found that virus-specific CD4^+^ T cells co-cultured with HSV-infected DCs previously treated with Dox (Figure 3I) or CoPP (Figure 3M) significantly expressed FoxP3 compared to the control DCs. Additionally, we assessed OX40 expression on the surface of these CD4^+^ T cells, which relates to activated Tregs [48]. Interestingly, OX40 was significantly more expressed in CD4^+^ T cells co-cultured with HSV-infected DCs previously treated with Dox (Figure 3D) or CoPP (Figure 3H). Finally, we also analyzed the levels of the cytokines IFN-γ, IL-4, and IL-17, which are mainly secreted by differentiated T cells (Th1, Th2 and Th17, respectively) to better characterize the phenotype of these T cells. While no significant changes were found for IFN-γ (Figure 3L,P) or IL-4 (Figure 3Q,S) with either inducer of HO-1, we observed a significant increase in IL-17 levels in the co-culture media, suggesting a Th17 phenotype for these cells (Figure 3R,T). Indeed, when we measured RAR-related orphan receptor gamma (RORγt) intracellular expression in T cells co-cultured with HSV-infected DCs induced to express HO-1, we found significantly increased expression of this transcription factor in contrast to the control conditions (Figure 3J,N). Interestingly, we also observed a mixed FoxP3/RORγt phenotype in a fraction of these cells (Figure 3K,O) that may relate to a transition between the Th17 and Tregs phenotypes [49].

Additionally, we assessed virus-specific CD8^+^ T cell activation (gBT-I CD8^+^ T cells) in co-cultures similar to those described above. In these cases, we did not observe a significant increase in the expression of IL-2, CD25, or CD71 in CD8^+^ T cells upon co-culture with DCs stimulated to express HO-1 and infected with HSV (Supplementary Figure S3).

### 3.4. Carbon Monoxide Recapitulates the Effects of HO-1 Expression in HSV-Infected DCs

**Figure 4:**
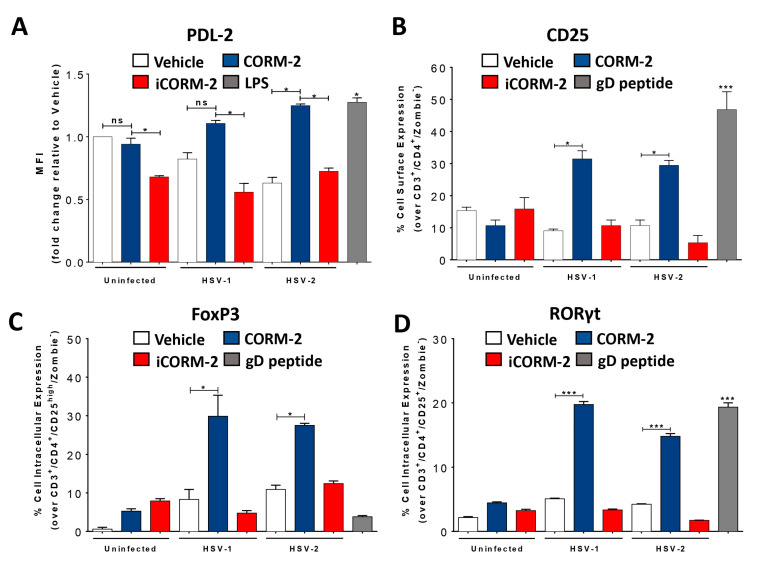
Carbon monoxide (CO) released by CORM-2 recapitulates HO-1 effects in HSV-infected DCs. (**A**) Surface expression of PDL-2 in DCs measured by FACS (in CD11c^+^, MHC-II (I-A^b+^), Zombie^-^ cells) at 24 hpi with HSV-1 or HSV-2, previously treated for 6 h with CORM-2 (60 µM). (**B**) Surface expression of CD25 in CD4^+^ T cells co-cultured with DCs, measured by FACS (in CD3^+^, CD4^+^, Zombie^-^ cells). (**C**) Intracellular expression of the transcription factor FoxP3 in CD4^+^ T cells by FACS (in CD3^+^, CD4^+^, CD25^high^, Zombie^-^ cells). (**D**) Intracellular expression of the transcription factor RORγt in CD4^+^ T cells by FACS (in CD3^+^, CD4^+^, CD25^high^, Zombie^-^ cells). Veh: Vehicle. iCORM-2: inactivated CORM-2. Statistical analysis: Kruskal–Wallis and Dunnett’s multiple comparison (* *p* < 0.05, *** *p* < 0.001).

Because the findings described above, regarding HO-1 expression in HSV-infected DCs should relate to one or more of the products of this enzyme, we sought to evaluate whether carbon monoxide (CO), which was previously found to recapitulate the anti-HSV effects of HO-1 in epithelial cells and neurons [22], also had a similar effect in these cells. DCs treated with CORM-2, a CO-releasing molecule, prior to HSV infection elicited comparable effects to CoPP treatment in terms of the surface expression of PDL-2 in these cells (Figure 4A). Consistent with a specific effect caused by CO, treating these cells with inactivated CORM-2 (iCORM-2) restored the levels of PDL-2 expression, similar to control cells (Figure 4A).

Because the induction of HO-1 expression in HSV-infected DCs with CoPP promoted the activation of CD4^+^ T cells and elicited both Treg and Th17 phenotypes, we assessed if treating these cells with CORM-2 also promoted these effects. Interestingly, virus-specific CD4^+^ T cells co-cultured with CORM-2-treated HSV-infected DCs were activated, and displayed increased surface expression of CD25 (Figure 4B) and expressed significantly more intracellular FoxP3 (Treg) and RORγt (Th17) than control cells (Figure 4C,D). Overall, these results suggest that the main effects mediated by the stimulation of HO-1 expression in HSV-infected DCs can be recapitulated by the HO-1 enzymatic product CO.

### 3.5. HO-1 Induction in HSV-Infected DCs Transferred into Mice Reduces Disease Severity and Modulates Virus-Specific T Cell Infiltration into HSV-1-Related Skin Lesions

**Figure 5:**
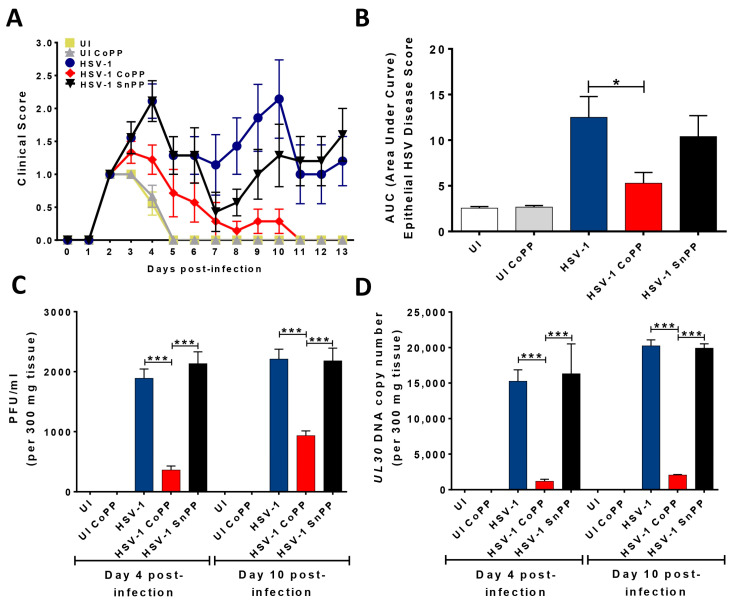
HSV-1 skin infection disease severity after the transfer of HSV-infected DCs treated to express HO-1 and later infected with HSV-1. (**A**) Disease scores in C57BL/6J animals infected dermally with 10^6^ PFU of HSV-1 24 h after the transfer of 10^6^ bone-marrow-derived HSV-infected DCs treated with CoPP, SnPP, or vehicle. (**B**) Area under the curve (AUC) values, which integrate disease score and duration. (**C**) Viral particles detected in an area of 1 cm^2^ around HSV-1 KOS- or mock-treated skin samples at days 4 and 10 after virus inoculation. Mice were previously transferred (1 day prior), with 10^6^ HSV-infected DCs treated with CoPP, SnPP, or vehicle. (**D**) HSV-1 genome copies (UL30 gene, qPCR) assessed in total DNA samples obtained from skin sections at 4 and 10 days after skin infection with HSV-1 KOS. One-way ANOVA with Tukey’s multiple comparison test was used for statistical analyses (* *p* < 0.05, *** *p* < 0.001). Data are means ± SEM (*n* = 5 mice/group).

To extend our understanding on the effects of the stimulation of HO-1 expression in DCs upon HSV infection, we assessed the contribution of these cells in a clinically relevant HSV-1 skin infection model [38,39]. For this, we followed the disease progression in animals receiving CoPP-treated HSV-infected DCs before dermal infection with HSV-1. As shown in Figure 5, mice receiving CoPP-treated HSV-infected DCs displayed reduced HSV-associated symptoms, delayed disease onset, as well as both reduced the maximum clinical scores and decreased the mean clinical scores at days 4 and 10 after infection (Figure 5A and Supplementary Table S1). Furthermore, animals that received CoPP-treated HSV-infected DCs prior to HSV-1 infection displayed significantly less skin pathology scores associated with HSV compared to mice receiving vehicle-treated HSV-infected DCs that were later infected with HSV in the skin, as determined by measuring the area under the curve (AUC) in Figure 5A, which provides an integrated value of score severity with disease duration (Figure 5B). In addition, we observed that transferring HSV-infected DCs induced to express HO-1 after CoPP treatment into the animals, and not vehicle-treated HSV-infected DCs or SnPP-treated HSV-infected DCs, significantly reduced HSV-1 loads in the infected tissue (Figure 5C), as both lesser PFUs and viral genome copies were detected in the skin samples recovered (Figure 5D).

**Figure 6:**
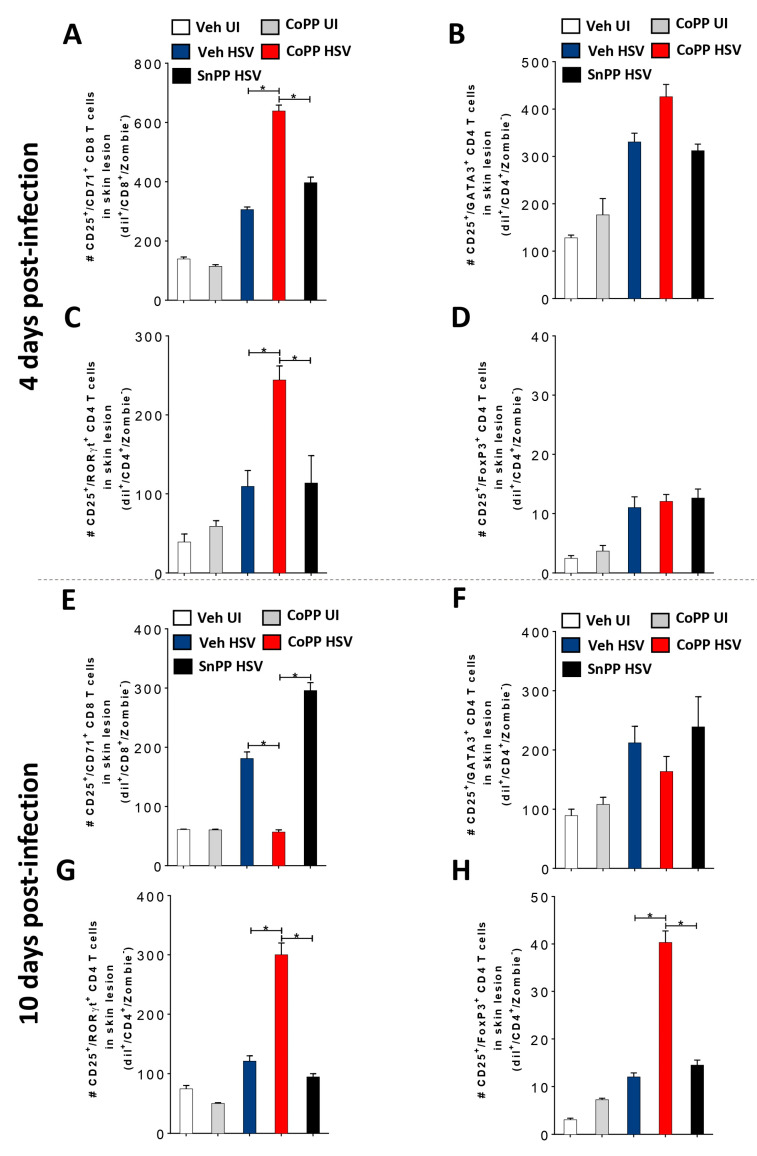
Phenotype and dynamics of virus-specific T cells infiltrating HSV-1-infected skin lesions after the transfer of HSV-infected DCs induced to express HO-1 and later HSV-1 infection. (**A**) Quantification of activated transgenic gBT-I virus-specific CD8^+^ T cells (CD25^+^/CD71^+^/Zombie^-^ cells) tracked with the dye Dil (Dil^+^/CD8^+^) in skin lesions (1 cm^2^) at day 4 and day 10 (**E**) after HSV-1 KOS skin infection in C57BL/6J mice that were previously injected (2 days prior to infection) with transgenic virus-specific gBT-I CD8^+^ T cells together with transgenic virus-specific gDT-II CD4^+^ T cells, and then treated 24 h later with HSV-1-infected DCs treated with vehicle, CoPP, or SnPP (1 day prior to infection). (**B**) Quantification of activated virus-specific activated (CD25^+^/CD71^+^/Zombie^-^) gDT-II CD4^+^ T cells with a Th2 phenotype (GATA3^+^) tracked with the dye Dil (Dil^+^/CD4^+^) in skin lesions at day 4 and day 10 (**F**) after HSV-1 infection in mice treated similarly as in (**A**). (**C**) Quantification of activated transgenic virus-specific gDT-II CD4^+^ T cells with a Th17 phenotype (RORγt^+^) in skin lesions at day 4 and day 10 (**G**) after HSV-1 infection in mice treated similarly as in (**A**). (**D**) Quantification of activated transgenic virus-specific activated (CD25^+^/CD71^+^/Zombie^-^) gDT-II CD4^+^ T cells with a Treg phenotype (FoxP3^+^) in skin lesions at day 4 and day 10 (**H**) after HSV-1 infection in mice treated similarly as in (A). One-way ANOVA with Tukey’s multiple comparison test was used for statistical analyses (* *p* < 0.05). Data are means ± SEM (*n* = 5 mice/group for skin lesion determination).

Next, we analyzed the amounts and phenotypes of HSV-specific T cells infiltrating virus-induced skin lesions 4 and 10 days after HSV-1 skin infection in mice that had previously received the treated DCs. Prior to receiving the treated DCs and being infected, these mice were transferred with untouched purified naïve transgenic gBT-I CD8^+^ and gDT-II CD4^+^ T cells, in order to follow virus-specific T cell populations in the HSV-infected skin after challenge. As shown in Figure 6, by day 4 post-infection, we detected activated virus-specific CD8^+^ (Figure 6A) and CD4^+^ T cells in the infected skin, with most gDT-II CD4^+^ T cells displaying Th2 (GATA3^+^) (Figure 6B) and Th17 (RORγt^+^) (Figure 6C) phenotypes. However, mice receiving the DCs induced to express HO-1 with CoPP displayed a significant increase in Th17 cells compared to the animals receiving non-treated or SnPP-treated HSV-infected DCs (Figure 6C). Although other gDT-II CD4^+^ T cell phenotypes, such as Treg (Figure 6D), were detected in the skin at this time-point, they were poorly represented within total virus-specific CD4^+^ T cells and did not significantly vary among the groups transferred with the differentially treated HSV-infected DCs.

Interestingly, by day 10 post-infection, the amount of activated virus-specific CD8^+^ T cells infiltrating the skin of animals that received DCs induced to express HO-1 with CoPP significantly decreased compared to the previous time-point and was even lower than the levels detected in animals receiving non-treated HSV-infected DCs (Figure 6E). Again, at this time-point, most of the virus-specific CD4^+^ T cells infiltrating the infected skin had Th2 (Figure 6F) and Th17 (Figure 6G) phenotypes, and virus-specific Th17 cells were again significantly increased in mice receiving DCs induced to express HO-1 with CoPP, as compared to the other treatments (Figure 6G). Noteworthy, by day 10 post-infection, Tregs infiltrating the skin were now also significantly increased in this group, evidencing significant temporal and phenotypical dynamics of virus-specific CD4^+^ T cells infiltrating the infected skin in the different groups (Figure 6H). These results suggest that transferring DCs stimulated to express HO-1 promotes virus clearance, confers to T cells particular phenotypes compared to untreated HSV-infected DCs, and favors reduced HSV skin disease.

## 4. Discussion

Here, we observed that the viability of DCs infected with HSV was significantly increased when these cells were treated to express HO-1. Interestingly, HO-1 expression in DCs infected with HSV did not significantly promote their maturation, which is consistent with prior studies reporting that HO-1 expression in DCs promotes a tolerogenic phenotype with reduced maturation, which can dampen antigen-specific immune responses [50,51]. Indeed, some studies report that HO-1 expression promotes the expression of co-inhibitory molecules, such as PDL-1 and PDL-2, on the DC surface, similar to what we observed herein [47,52]. However, the T cell-activating capacity of HO-1-expressing DCs may differ in the context of viral infections, such as with HSV, as this virus encodes molecular determinants that are recognized by pathogen recognition receptors (PRRs) expressed in DCs, which will likely elicit signaling cascades within HO-1-expressing infected cells that can modulate the overall phenotype of these DCs [53,54,55]. Additionally, other reports have shown that reduced maturation markers on the surface of HSV-infected DCs does not preclude effective T cell activation [13,21].

Although DCs stimulated to express HO-1 and then infected with HSV did not significantly activate virus-specific CD8^+^ T cells in vitro, which is consistent with a previous report in a similar context but with the respiratory syncytial virus [27], this was not the case for virus-specific CD4^+^ T cells, which displayed an activated phenotype that was in stark contrast with that of CD4^+^ T cells co-cultured with HSV-infected DCs that were not treated to express HO-1 [12]. DCs treated to express HO-1 and then infected with HSV mainly promoted Treg and Th17 phenotypes in vitro. Noteworthily, previous studies report that HO-1-expressing DCs can promote anti-inflammatory T cell responses [52], yet the Th17 phenotype observed herein was unexpected. Although HO-1 stimulation with CoPP has been reported to upregulate the transcription factor Nrf-2 (nuclear factor erythroid 2-related factor 2) [28], which relates to the downregulation of NF-κB signaling, which in turn is required for events leading to DC profiles that induce Th17 phenotypes in CD4^+^ T cells [56,57,58], the fact that HSV significantly modulates NF-κB activation during infection could relate to a pro-Th17 T cell phenotype elicited in CD4^+^ T cells by HSV-infected DCs, despite HO-1 expression [4]. Overall, defining the factors that confer HO-1-expressing and HSV-infected DCs to promote the CD4^+^ T cell phenotypes observed herein deserves further analysis.

Additionally, we found that HO-1 induction with Dox treatment in DCs infected with HSV significantly increased their viability and inhibited virion release, as well as promoted an increased secretion of IL-10 and IL-23, which could modulate virus-specific CD4^+^ T cell differentiation in the co-cultures. Noteworthily, there are reports that indicate that tetracyclines, such as doxycycline, can have anti-apoptotic effects and reduce caspase-1 and caspase-3 activation [59], which could contribute to the increased cell viability observed herein in Dox-treated DCs infected with HSV. However, because DC treatment with CoPP elicited a similar effect over HSV-infected DCs, the increased viability of HSV-infected DCs observed in our study most likely is associated with HO-1 induction rather than the unspecific effects of doxycycline over these cells. A similar conclusion can be made regarding reports that indicate that doxycycline inhibits NF-κB [60], which could partially relate to the Th17 phenotype observed herein in virus-specific CD4^+^ T cells. However, again, similar results were obtained when using CoPP to induce HO-1 in DCs, indicating that the phenotype observed in our study most likely relates to HO-1 expression and not effects related to the treatment with doxycycline per se.

When analyzing the effects that HO-1 expression exerts over the replication cycle of HSV in infected DCs, we found that the expression of this host factor inhibited viral particle release into the supernatants and that an important fraction of infectious virus was cell-associated. This finding is consistent with the observation that HO-1 expression in HSV-infected DCs did not alter viral genome replication, the transcription of viral genes, such as *ICP0,* or the expression of a late viral protein (gB). However, these results contrast the mechanism by which HO-1 expression was found to interfere with the replication cycle of HSV-2 in epithelial cells, which was related to a blockade in HSV-2 capsid migration to the nucleus in infected cells [22]. Although we did not deepen on the molecular mechanisms impairing effective virion release into the supernatants in the current study, it is possible that the observed reduction in virus yield in the supernatants of HO-1-expressing and HSV-infected DCs measured at the indicated time-point herein may simply relate to a delay in the replication cycle of this virus in these DCs, which warrants further analysis.

Additionally, we found that carbon monoxide (CO) recapitulated relevant anti-HSV effects that are also mediated by HO-1 expression in HSV-infected DCs. Other studies also support the role of CO as an HO-1 product mediating significant effects by this enzyme [22]. Among others, this molecule has been reported to act as a signaling determinant that affects DC endosomal maturation and antigen presentation by uncoupling energy production that is dependent on the mitochondria [51]. Furthermore, other CO-mediated effects include a predisposition for smaller peptides being processed and loaded onto MHC molecules [61,62], and cellular energetic restrictions that promote co-inhibitory molecule expression [47].

Other studies have reported protective effects related to HO-1 expression after the exogenous administration of CORM in different experimental models of autoimmune diseases, including multiple sclerosis [63], type-1 diabetes [64], and autoimmune hepatitis [65]. The latter study, showed a positive effect upon CORM-A1 (a CO-releasing molecule) administration against mouse liver injury in a concanavalin-A-induced acute hepatitis model, which improved clinical and histological parameters [65]. On the other hand, prophylactic administration of CORM-A1 has been reported to reduce both clinical and histopathological signs of experimental autoimmune encephalomyelitis in mice, an animal model of multiple sclerosis [63]. Similarly, the impact of CORM-A1 on the development of diabetes has been evaluated in murine models, with CORM-A1 decreasing the incidence of diabetes in NOD mice, which was associated with an attenuated activity of M1 macrophages and the induction of CD4^+^ T cell differentiation toward a Th2 phenotype instead of Th1 or Th17 T helper cells [64]. Overall, these findings are consistent with those reported by Yan and collaborators, wherein pretreatment with CORM-3 (Tricarbonylchloroglycinatoruthenium(II), another CO-releasing molecule) reduced liver injury induced by lipopolysaccharide/D-galactosamine in a mouse model and increased the survival rate of the treated mice compared to controls [66].

Skin infected with HSV-1 after the transfer of DCs that were treated ex vivo to express HO-1 and HSV infection displayed reduced disease severity, a decrease in duration, and infiltrating virus-specific T cells as the disease progressed. Although HO-1 expression in HSV-infected DCs did not promote virus-specific CD8^+^ T cell activation in vitro, nevertheless, we observed a significant infiltration of virus-specific CD8^+^ T cells in the skin upon HSV infection at 4 days post-infection. It will be interesting to determine the factors that promote the activation of these virus-specific CD8^+^ T cells in vivo versus in vitro and to assess the dynamics of T cells infiltrating the skin, as well as the contribution of these cells to HSV skin disease resolution, particularly given the fact that these cells were practically no longer present at day 10 post-infection in this tissue. This result may provide a perspective of early effector T cell activity in this infected tissue and their potential contribution to viral control and to limiting herpes simplex zosteriform symptoms in the skin [67]. Noteworthily, in some chronic pathologies, such as atopic dermatitis, the sustained presence of CD8^+^ T cells in the skin increases the risk of HSV-1 *eczema herpeticum* [68].

The transfer of HO-1-expressing and HSV-infected DCs into mice later infected with HSV-1 in the skin also promoted shifts in virus-specific Th cell populations in this tissue as infection progressed. While the preponderant Th phenotypes of virus-specific CD4^+^ T cells infiltrating this tissue at day 4 and 10 were Th17 and Th2 under normal circumstances, differences regarding the magnitude of these and other phenotypes occurred upon the stimulation of HO-1 expression in HSV-1-infected DCs. For instance, Th17 cells were significantly increased in the skin lesions at day 4 and 10 post-infection in mice receiving HO-1-expressing and HSV-infected DCs. To our knowledge, HO-1 expression in DCs has not been related to the promotion of a Th17 phenotype in CD4^+^ T cells; yet again, this may be a particular phenomenon related to viral infections, and more specifically, to HSV infection, that has been scarcely studied [27,69]. Since Th17 cells have been reported to be plastic in terms of their capacity of acquiring features associated with other Th phenotypes, such as those related to Th1 cells or Tregs, the implications of our findings could relate to HO-1 expression in DCs, conferring these cells to have the ability to fine-tune Th responses with favorable outcomes regarding HSV infection [70,71]. Interestingly, we observed mixed populations of T cells including CD4^+^ T cells that were both RORγt- and FoxP3-positive, which has been related to a trans-differentiation state between Tregs and Th17 cells [49,72,73].

Although Treg were not necessarily the most prevalent Th phenotype in the skin at day 10 post-infection, there was a significant increase in this population in mice receiving HO-1-induced and HSV-infected DCs. Noteworthily, the presence of Treg in this tissue may help prevent the otherwise deleterious effects of Th2 cells, as reported in other contexts [74]. Nevertheless, the effects of different Th phenotypes in the skin is complex, with one study reporting that Treg depletion decreases the severity of HSV-2 skin lesions, implying that their presence could be detrimental for the host after an initial exacerbated inflammation [75]. However, in the same study, the adequate resolution of a skin infection with a thymidine kinase (TK)-deficient HSV-2 mutant was correlated with an early increase in Th17 cells, followed by a later appearance of Tregs, similar to what we observed herein in vivo.

Dimethyl fumarate (DMF, Tecfidera^®^), is a fumaric acid ester derivative approved by the Food and Drug Administration (FDA) of the United States to treat relapsing-remitting multiple sclerosis and psoriasis and displays significant immune-modulatory and antioxidant effects [76,77]. DMF is also an inducer of HO-1 and Nrf2 [78,79]. Importantly, DMF has been reported to suppress HIV replication and neurotoxin release through the inhibition of the nuclear translocation of NF-κB together with the induction of HO-1 expression [80]. Because our study overall showed a favorable immune response against HSV-1 skin disease when inducing HO-1 in DCs, it will be interesting to assess whether DMF treatment can recapitulate the findings reported herein. Importantly, a previous study found that DMF improved herpetic keratitis by increasing IL-4 and IL-5 secretion, both Th2-related cytokines, by activated lymphocytes [81]. Moreover, another research group recently reported antiviral effects for DMF against HSV infections in vitro, reducing viral yields both at 1 h and 4 h post-infection [82]. Notably, this study suggests the possibility of using a DMF-loaded gel composed of ethosomes for the topical treatment of HSV-1 infections, which remains to be assessed [82]. Importantly, an in vivo irritation test demonstrated that this gel is safe to apply over intact human skin.

## 5. Conclusions

Taken together, the findings described herein suggest a relevant role for HO-1 in DCs in the context of HSV infection, as the expression of this host factor significantly increased the viability of these cells and improved disease outcome after HSV infection. Furthermore, the expression of HO-1 in DCs significantly altered the dynamics of skin lesion virus-specific T cells and their phenotypes, which likely had an impact on overall skin disease outcome. Finally, our results suggest that the HO-1 enzymatic product CO may be sufficient for potentiating these anti-HSV effects in DCs and, thus, may prove as a potentially new therapeutic approach against HSV infection.

## Data Availability

Data is contained within the article or supplementary material. The data presented in this study are available in the figures and supplementary materials. Further inquiries can be directed to the corresponding author.

## Supplementary Materials

The following supporting information can be downloaded at: https://www.mdpi.com/article/10.3390/antiox12061170/s1, Figure S1: HO-1 induction in infected DCs by HSV-1 or HSV-2 does not affect viral replication or transcription; Figure S2: HO-1 does not promote significant maturation in HSV-infected DCs; Figure S3: HO-1 expression in HSV-infected DCs does not promote CD8^+^ T cell activation in vitro; Figure S4: HO-1 expression in HSV-infected DCs upregulates FoxP3 expression in CD4^+^ T cell in vitro; Figure S5: Gating strategy to determine the populations of virus-specific CD4^+^ T cells in tissues recovered after HSV-1 skin infection in mice transferred with HO-1-expressing and HSV-infected DCs in vitro; Table S1: Clinical score and disease symptom incidence in HSV-1 skin-infected mice transferred with HO-1-expressing and HSV-infected DCs.
