# Supplementary material for: Heme Oxygenase-1 Expression in Dendritic Cells Contributes to Protective Immunity against Herpes Simplex Virus Skin Infection
Source: Antioxidants (Basel). 2023 May 29;12(6):1170. doi: 10.3390/antiox12061170 (PMC10294886; doi:10.3390/antiox12061170)
Supplement: Supplementary file 1 [file antioxidants-12-01170-s001.zip › antioxidants-2321689-supplementary.pdf]

SUPPLEMENTARY MATERIAL

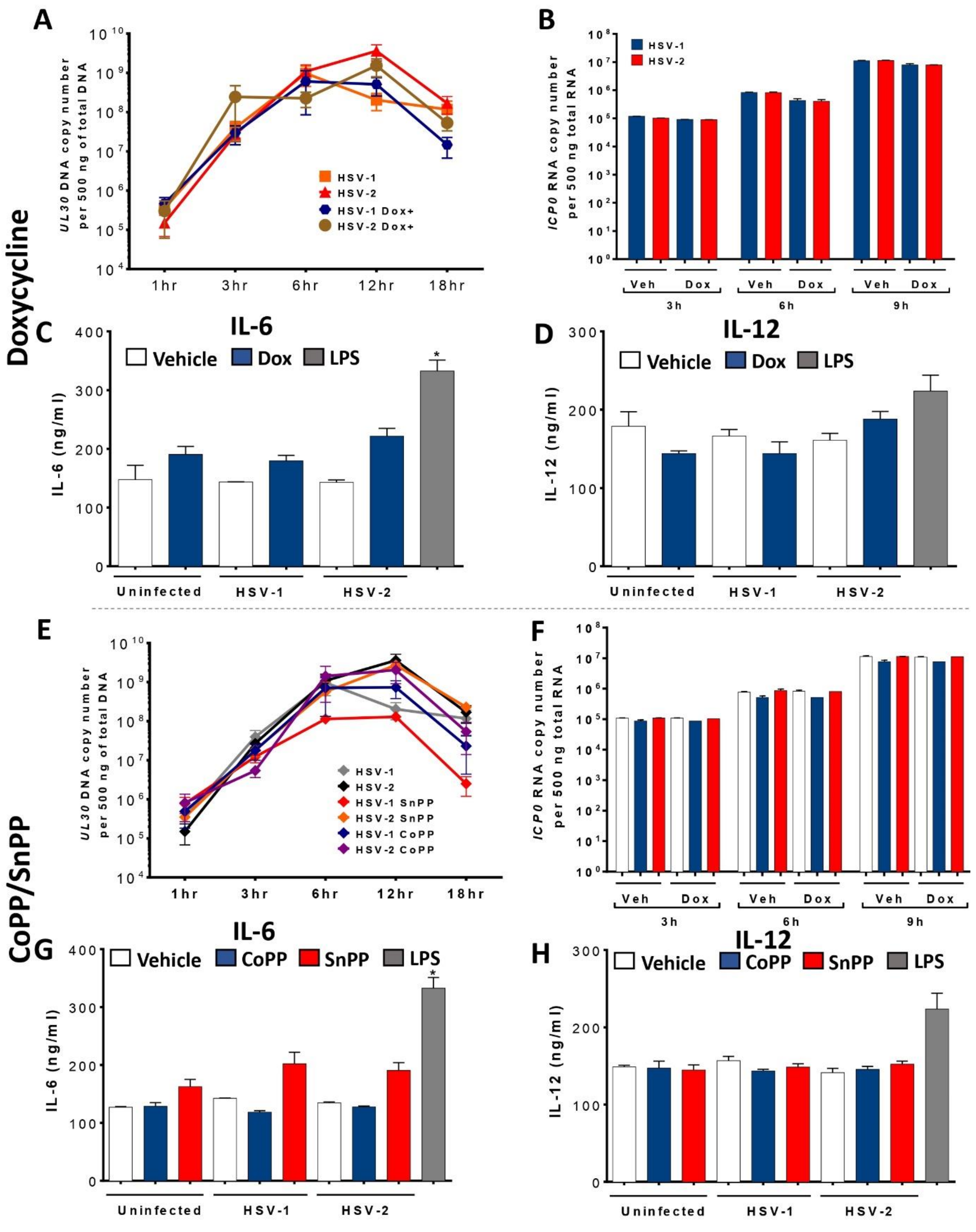

Supplementary Figure S1. HO-1 induction in infected DCs by HSV-1 or HSV-2 does not affect viral replication or transcription. (A) Copy number of HSV-1 or HSV-2 UL30 (qPCR) in total DNA obtained from Dox-treated DCs and infected HSV-1 or HSV-2 at timepoints 3, 6, 9, 12 and 18 hpi. (B) Copy number of HSV-1 or HSV-2 *ICP0* (RT-qPCR) measured in Dox-treated DCs after 6 h of HSV-1 or HSV-2 infection. (C) IL-6 secretion in Dox-treated HSV-infected DCs at 24 hpi. (D) IL-12 secretion in Dox-treated HSV-infected DCs at 24 hpi. (E) Copy number of HSV-1 or HSV-2 UL30 (qPCR) in total DNA obtained from CoPP- or SnPP-treated DCs and infected HSV-1 or HSV-2 at timepoints 3, 6, 9, 12 and 18 hpi. (F) Copy number of HSV-1 or HSV-2 *ICP0* (RT-qPCR) measured in CoPP- or SnPP-treated DCs after 6 h of HSV-1 or HSV-2 infection. (G) IL-6 secretion in CoPP- or SnPP-treated HSV-infected DCs at 24 hpi. (H) IL-12 secretion in CoPP- or SnPP-treated HSV-infected DCs at 24 hpi. LPS: lipopolysaccharides. Statistical analysis: Kruskal–Wallis and Dunnett’s multiple comparison (*p <0.05).

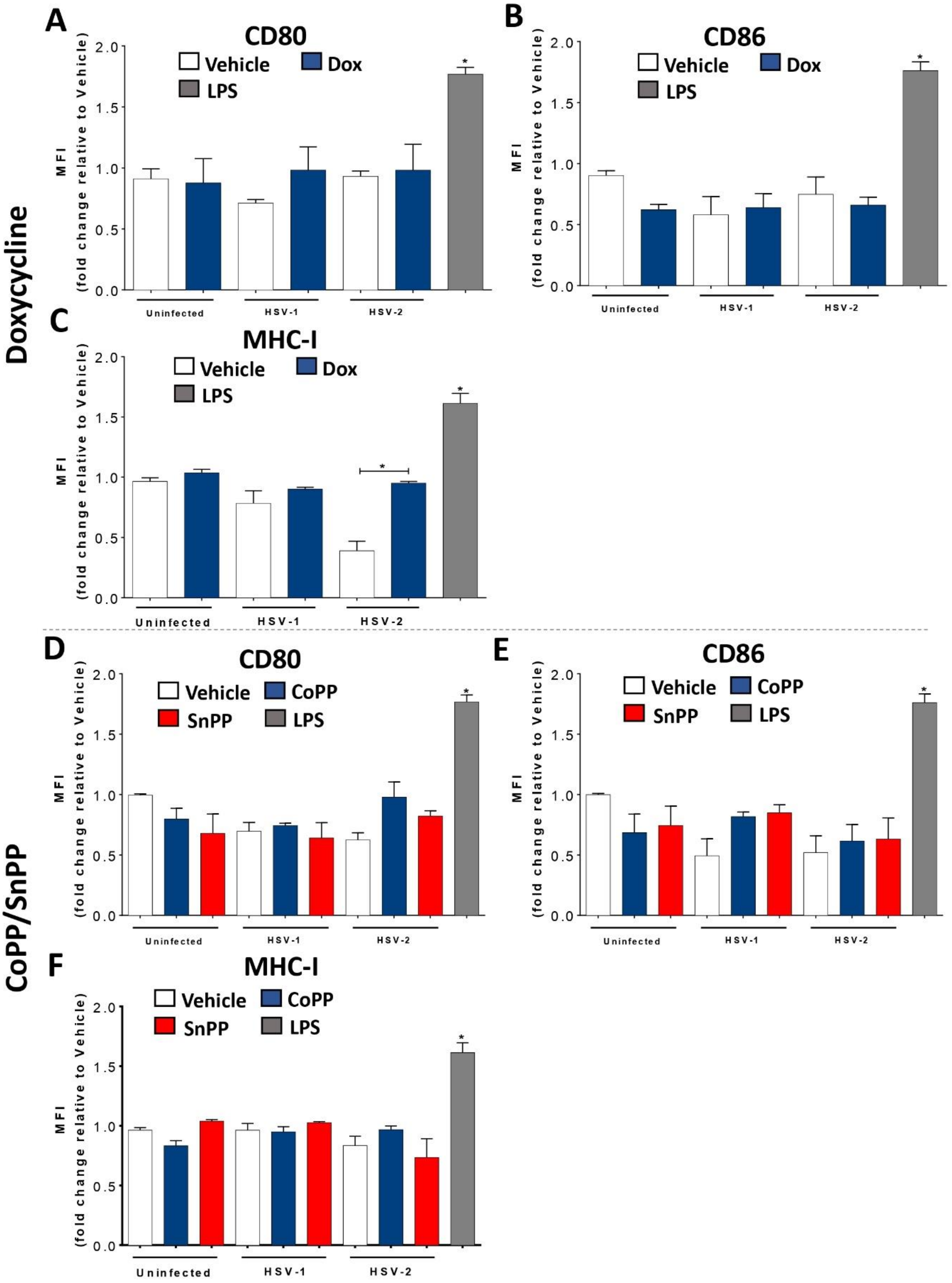

Supplementary Figure S2. HO-1 does not promote significant maturation in HSV-infected DCs. (A) Surface expression of CD80 in DCs measured by FACS (over CD11c$^+$, MHC-II$^+$ (I-Ab$^+$), Zombie$^-$) at 24 hpi with HSV-1 or HSV-2 after 16 h treatment

with doxycycline (Dox, 1.5 μg/ml). (B) Surface expression of CD86 in DCs. (C) Surface expression of MHC-I (H-2K^b) in DCs. (D) Surface expression of CD80 in DCs as measured by FACS (over CD11c^+, MHC-II (I-Ab^+), $Zombie^-$) at 24 hpi with HSV-1 or HSV-2 after treatment with CoPP or SnPP (50 μM). (E) Surface expression of CD86 in DCs. (F) Surface expression of MHC-I (H-2K^b) in DCs. LPS: lipopolysaccharides. Statistical analysis: Kruskal–Wallis and Dunnett's multiple comparison (*p <0.05; ***p <0.001).

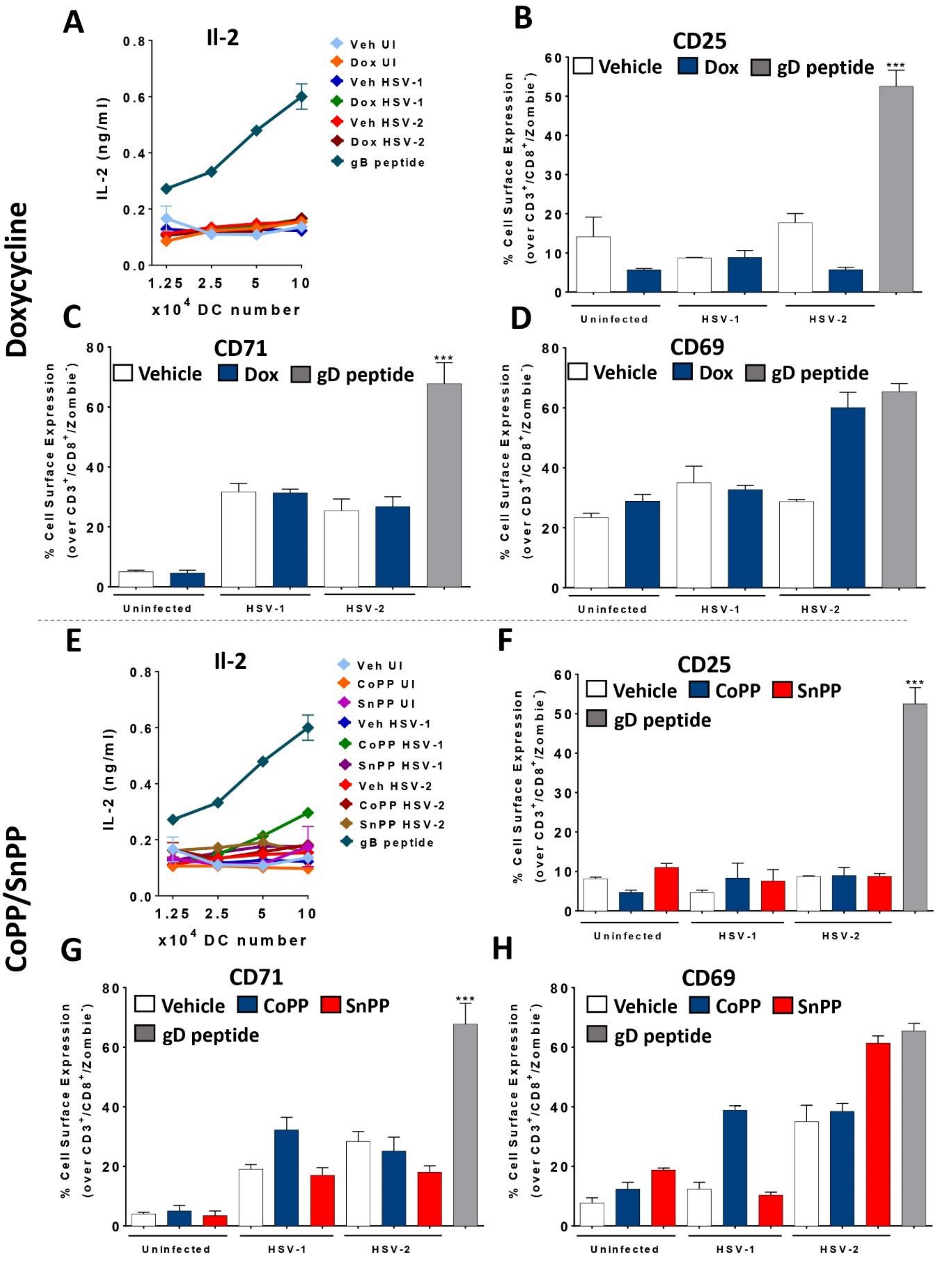

Supplementary Figure S3. HO-1 expression in HSV-infected DCs does not promote CD8+ T cell activation *in vitro*. (A) Secretion levels of IL-2 in supernatants from co-cultures between CD8 T and HSV-infected and treated DCs as measured by ELISA

at 48 hpi with HSV-1 or HSV-2 after 6 h treatment with CoPP or SnPP (50 μM). (B) Surface expression of CD25 in CD8 T cells stimulated by DCs, measured by FACS (over $CD3^{+}$, $CD8^{+}$, $Zombie^{-}$) with its respective treatment conditions. (C) Surface expression of CD71 in CD8 T cells (Dox). (D) Surface expression of CD69 in CD8 T cells (Dox). (E) Secretion levels of IL-2 in supernatants from CD4 T cells co-cultured with DCs after 6 h treatment with CoPP or SnPP (50 μM). (F) Surface expression of CD25 in CD8 T cells co-cultured with DCs after 6 h treatment with CoPP or SnPP (50 μM). (G) Surface expression of CD71 in CD8 T cells co-cultured with DCs after 6 h treatment with CoPP or SnPP (50 μM). (H) Surface expression of CD69 in CD8 T co-cultured with DCs after 6 h treatment with CoPP or SnPP (50 μM). Statistical analysis: Kruskal–Wallis and Dunnett's multiple comparison (*p <0.05, ***p < 0.001).

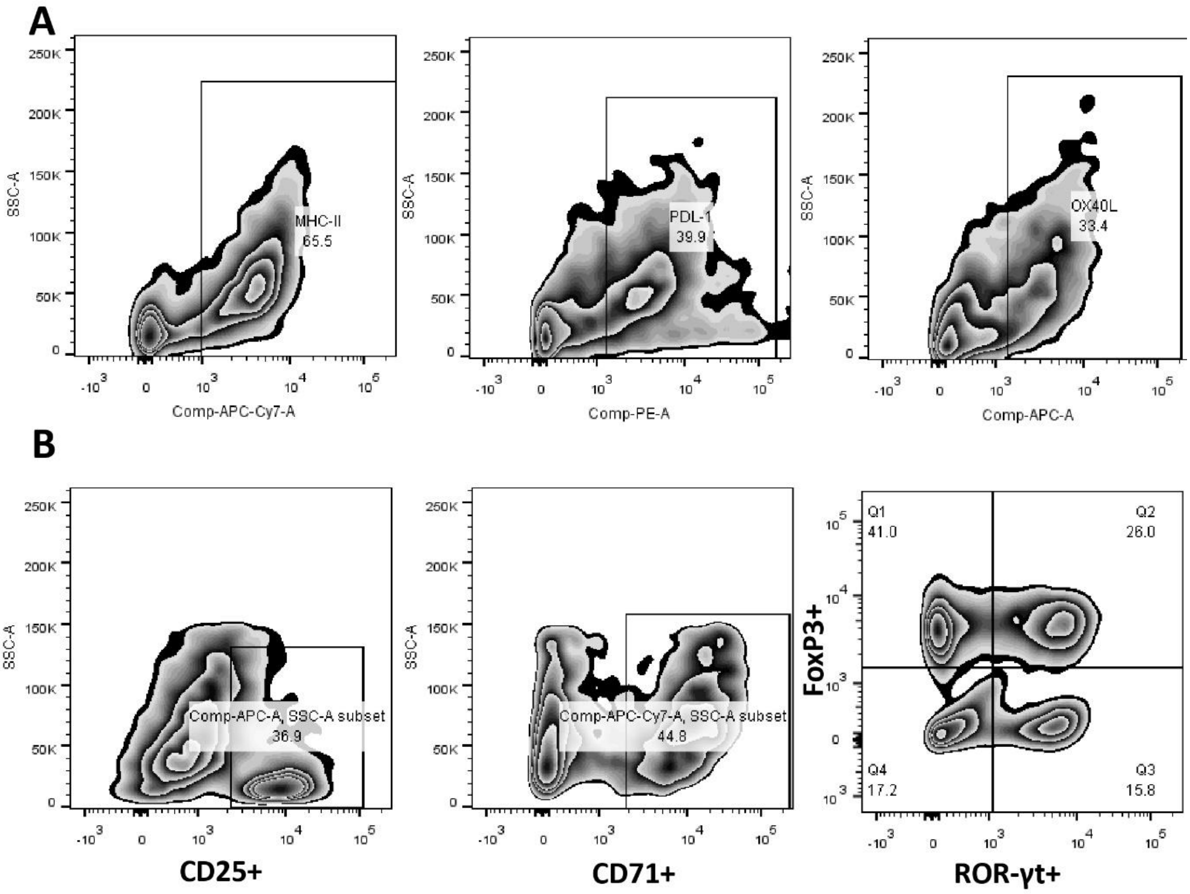

Supplementary Figure S4. HO-1 expression in HSV-infected DCs upregulates FoxP3 expression in CD4$^+$ T cell *in vitro.* (A) Representative zebra blots of surface markers in DCs analyzed by FACS. (B) Representative zebra blots of surface, and intracellular markers in CD4$^+$ T cells analyzed by FACS.

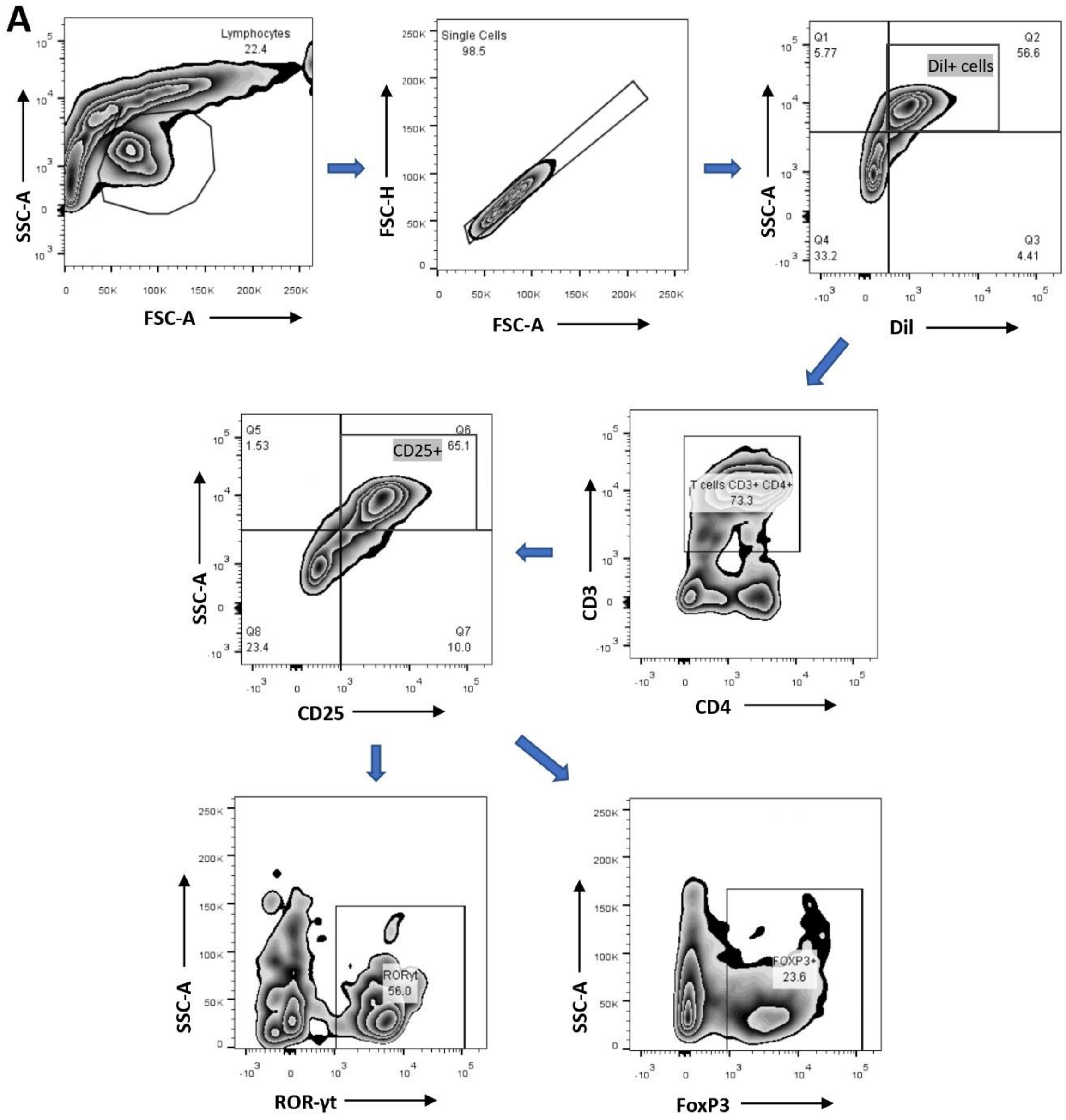

Supplementary Figure S5. Gating strategy to determine the populations of virus-specific CD4^{+} T cells in tissues recovered after HSV-1 skin infection in mice transferred with HO-1-expressing and HSV-infected DCs *in vitro*.

Supplementary Table S1. Clinical score and disease symptom incidence in HSV-1 skin-infected mice transferred with HO-1-expressing and HSV-infected DCs.

Group	Incidence of HSV-derived Symptoms*	Mean Day of Disease Onset	Maximum Clinical Score	Mean Clinical Score at Day 4	Mean Clinical Score at Day 10
UI	0% (0/9)	0	1	0.55	0
UI CoPP	0% (0/9)	0	1	0.66	0
HSV-1 KOS	100% (9/9)	3	4	2.11	2.14
HSV-1 CoPP	33.3% (3/9)	4	2	1.22	0.28
HSV-1 SnPP	77.7% (7/9)	3	3	2.11	1.28

* Scores from 2 and above relate to disease elicited by skin HSV infection.
